# A method to incorporate prior information into score test for genetic association studies

**DOI:** 10.1186/1471-2105-15-24

**Published:** 2014-01-22

**Authors:** Sergii Zakharov, Garrett HK Teoh, Agus Salim, Anbupalam Thalamuthu

**Affiliations:** 1 Human Genetics, Genome Institute of Singapore, 60 Biopolis Street, #02-01 Genome, Singapore 138672, Singapore; 2 Saw Swee Hock School of Public Health, National University of Singapore, 16 Medical Drive, Singapore 117597, Singapore; 3 Department of Mathematics and Statistics, La Trobe University, Bundoora, VIC 3086, Australia; 4 Centre for Healthy Brain Ageing (CHeBA), School of Psychiatry, University of New South Wales, Sydney, Australia

**Keywords:** Prior information, Association analysis, Score test, Rare variants

## Abstract

**Background:**

The interest of the scientific community in investigating the impact of rare variants on complex traits has stimulated the development of novel statistical methodologies for association studies. The fact that many of the recently proposed methods for association studies suffer from low power to identify a genetic association motivates the incorporation of prior knowledge into statistical tests.

**Results:**

In this article we propose a methodology to incorporate prior information into the region-based score test. Within our framework prior information is used to partition variants within a region into several groups, following which asymptotically independent group statistics are constructed and then combined into a global test statistic. Under the null hypothesis the distribution of our test statistic has lower degrees of freedom compared with those of the region-based score statistic. Theoretical power comparison, population genetics simulations and results from analysis of the GAW17 sequencing data set suggest that under some scenarios our method may perform as well as or outperform the score test and other competing methods.

**Conclusions:**

An approach which uses prior information to improve the power of the region-based score test is proposed. Theoretical power comparison, population genetics simulations and the results of GAW17 data analysis showed that for some scenarios power of our method is on the level with or higher than those of the score test and other methods.

## Background

In spite of the success of genome-wide association studies (GWAS) in identifying hundreds of common single nucleotide polymorphisms (SNPs) associated with diseases and complex traits (http://www.genome.gov/gwastudies/), in many cases the proportion of heritability explained by these discovered SNPs is low [1-3]. One of the potential explanations of this observation is that rare variants (usually defined as those with minor allele frequency below 1%), which are absent from conventional GWAS studies, are responsible for the missing heritability. Indeed, there is strong evidence that rare variants are associated with some complex traits [4-8].

When performing rare variants association analysis researchers face significant methodological challenges. The single SNP approach, which is popular in GWA studies, is underpowered when applied in rare variants association studies with moderate sample size due to low allele count for each individual rare variant. To overcome this problem, testing multiple rare variants within a region has been recommended [9,10], and a number of region-based rare variants tests have been proposed [11-15]. However, these novel methodologies may still be underpowered for association studies with moderate sample size [16]. This motivates the development of statistical methods that utilize prior information with the purpose of improving power. Currently, much biological information is publicly available, such as the prediction of degree of deleteriousness of non-synonymous variants (PolyPhen http://genetics.bwh.harvard.edu/pph2/, SIFT http://sift.bii.a-star.edu.sg/), SNP prioritization (FastSNP http://fastsnp.ibms.sinica.edu.tw), functional SNP annotation (SNPnexus http://www.snp-nexus.org/) etc. Although several methods that use prior information have been proposed [17-20], further research is needed to utilize prior knowledge more efficiently [21] and to expand statistical tools available for researchers.

In this article we propose a method that incorporates prior information into the region-based score test. Within our framework, prior information is used to partition SNPs within a genomic region of interest into groups. Then within each group asymptotically independent SNP scores are combined into a one degree of freedom (d.f.) chi-squared statistics. These group statistics are then used to construct a global test for a region. The proposed methodology has several distinct advantages. First of all, the degrees of freedom of our test statistic equals to the number of groups, which may be much less than the number of SNPs within a region. Secondly, under partitioning that approximately separates associated variants from neutral ones (“informative” partitioning) the proposed method efficiently handles noise introduced by neutral variants. We have evaluated the performance of our method on theoretical power comparison, population genetics simulations and analysis of the GAW17 (http://www.gaworkshop.org/gaw17/) real sequencing data set. The results showed that under some scenarios the proposed methodology performed as well as or outperformed the score test and other competing methods.

## Results

### Theoretical power comparison

**Figure 1:**
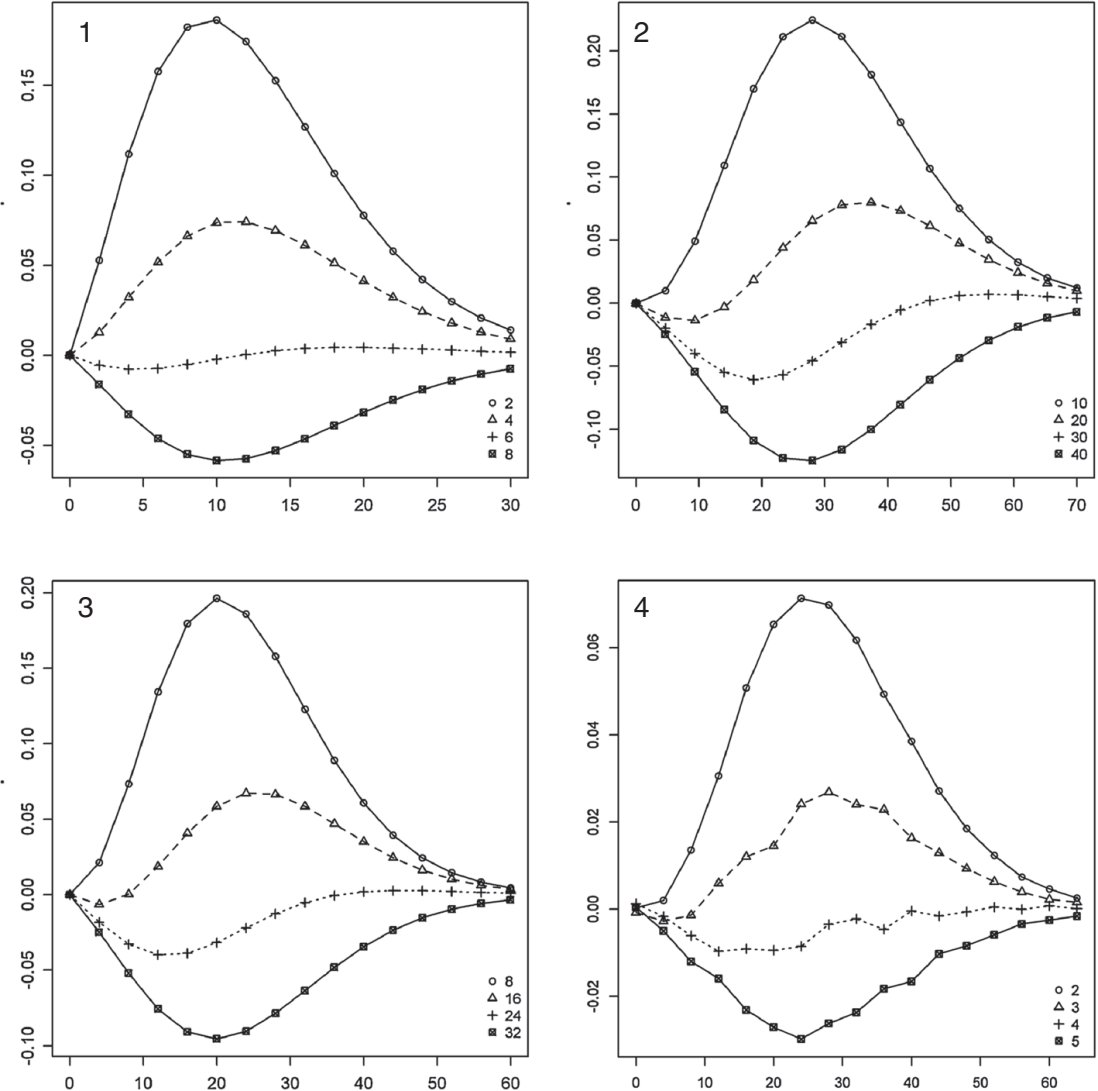
**The difference in theoretical power (vertical axis) of the proposed test and the score test as a function of the total non-centrality parameter *r *(horizontal axis) at the type-1 error rate *α*** **= 0.05.** Each curve corresponds to the number of SNPs in the single causal group *L*_1_ given in the legend (Panels 1 and 2), the number of groups *K* (Panel 3), and the number of causal groups *m* (Panel 4). The parameters for each of the Panels are as follows: Panel 1: *L* = 10, *K* = 2; Panel 2: *L* = 100, *K* = 10; Panel 3: *L* = 50, *L*_1_ = 5; Panel 4: *L* = 54, *K* = 6, equal number of SNPs in each group, and equal non-centrality parameters in all causal groups.

Figure 1 shows the difference between the theoretical power of the proposed method and those of the score test for different scenarios at the fixed type-1 error rate of 0.05. For Panel 1 of Figure 1, the number of SNPs within a region *L*=10, the number of groups in a partitioning *K*=2, the range of values for the non-centrality parameter (NCP) *r* 0–30 (x-axis), and the number of variants in a causal group *L*_1_=2,4,6,8 (different values of *L*_1_ correspond to different curves). As can be seen, the power gain of the proposed method reached as high as 15 percent when the number of SNPs in the causal group *L*_1_ was not large. However, the power of the proposed test was lower than those of the score test when *L*_1_ was large. This can be explained from the formula (3), which implies that with an increase in the number of variants in a causal group *L*_1_ and fixed NCP *r* the value of the group statistic *H*_1_ monotonically decreases. Also, it is noticeable that with an increase in NCP *r* up to some point the power difference also increased when *L*_1_ was small, whereas the power difference decreased for large values of *L*_1_. For very large values of NCP *r* the power difference became close to 0 as theoretical powers of both tests tended to 1. Similar conclusions can be obtained from Panel 2 of Figure 1 which shows the results for the scenario with the following parameters: *L*=100, *K*=10, varying NCP *r* (x-axis), and varying *L*_1_=10,20,30,40.

To investigate the impact of the number of groups *K* on power the following model was considered (Panel 3 of Figure 1): *L*=50, *L*_1_=5, and *K*=8,16,24,32. As can be seen, the power difference is below zero for large values of *K* and above zero for small values of *K*. This may be explained by the fact that the test statistic *T*_1_ (4) is distributed as chi-squared random variable with *K* d.f. Thus, for the fixed NCP *r* and increasing value of *K* the power of the proposed method monotonically decreases.

Alongside the models considered above, it is important to include a scenario when causal variants are split between several groups. Panel 4 of Figure 1 depicts the power difference under the following model: *L* = 54, *K* = 6, and the number of causal groups *m* = 2,3,4,5. Also, for simplicity of presentation it was assumed that each group contained equal number of variants (54/6 = 9), and NCP *r* was split equally between all causal groups. For this scenario the calculation of theoretical distribution of *T*_1_ (4) was done using 500,000 simulations of test statistics under the alternative hypothesis, as equation (6) cannot be used here. As can be seen from Panel 4 of Figure 1, our methodology could gain power if associated SNPs were split between several groups. When the number of causal groups became large, our method was slightly worse than the score test. It should be noted that for all models the maximum power gain of our method (and equivalently the maximum power loss) was achieved when the power of the score test was around 50%. Conclusions similar to those above can be derived from Additional file 1 which shows the same scenarios for the fixed type-1 error rate set at the genome-wide level assuming 35,000 genes (*α* = 0.05/35000).

### Population genetics simulations results

Using population genetics simulations we compared our method with the score test and other proposed approaches, namely, weighted selective collapsing strategy (WSCS) [22], variable threshold (VT) [18], weighted sum of squared scores test (SSUw) [23], and optimal sequence kernel association test (SKAT-O) with linear kernel and beta weights [24]. Since these tests utilize only genotype and phenotype information, we applied our method with MAF (minor allele frequency) partitioning: variants within a region are divided into two groups, namely, those with observed MAF above and below 1%. To estimate the empirical type-1 error we ran the analysis of simulated data under the null model of no association (Additional file 2). As can be seen from Additional file 2, the type-1 error was well controlled for all the tests.

**Figure 2:**
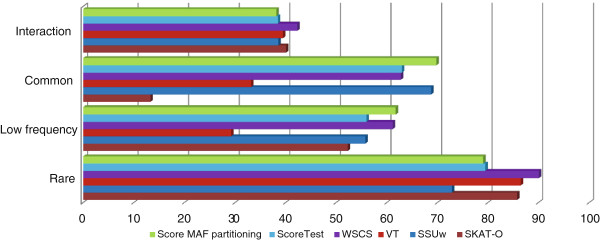
**Comparison of the proposed method with MAF partitioning and other statistical tests on population genetics simulations.** In the “Rare” phenotype model only rare variants (MAF<1% in haplotype pool) were causal with uniform effect size. “Low Frequency” and “Common” phenotype models had only one low frequency (MAF between 1% and 5%) and one common (MAF>5%) causal SNP respectively. Finally, the “Interaction” scenario models the interaction of rare variants with a common SNP. A minor allele of a rare causal variant had an impact on phenotype if and only if it was present on the same haplotype as a minor allele of a common SNP chosen beforehand.

Figure 2 shows the results of the population genetics simulations. As can be seen, our method was the most powerful for both “Low Frequency” and “Common” phenotype models, closely followed by WSCS and SSUw respectively. For the “Interaction” model WSCS achieved slightly higher power than other methods. For the “Rare” model WSCS and VT tests showed the highest power, whereas our method performed worse than most other methods. Although in this scenario all the causal variants had MAF < 1% in a haplotype pool, some of the causal variants were expected to have MAF > 1% in a data replicate, since causal alleles increased the probability of a disease. Thus, in this case applying MAF partitioning could result in causal variants with high MAF being combined with neutral common SNPs.

### GAW17 analysis results

In addition to theoretical power comparison and population genetics simulations we also used the GAW17 data set to compare our test with other methods. The GAW17 data set was designed to mimic a real exome sequencing study of a complex disease. Full description of the method used to generate the GAW17 data can be found in Almasy et al. [25]. Briefly, the whole-exome sequencing data from 1000 Genomes Project (http://www.1000genomes.org) was the basis for simulation of 200 replicates of dichotomous phenotype and 200 replicates of 3 quantitative traits (endophenotypes) in 697 unrelated individuals from six populations (Chinese, Japanese, Yoruba, Luhya, Utah residents with Northen and Western European ancestry – CEPH, and Tuscan). Causal variants were chosen to be common and rare non-synonymous SNPs concentrated in genes from specific pathways. Also, dichotomous phenotype and quantitative traits were impacted by covariates such as smoking status, gender and age.

We conducted an association analysis of known causal genes with two quantitative traits (third quantitative trait was simulated independently from genotype) and a dichotomous phenotype adjusting for covariates and population stratification. The adjustment procedure was similar to those described by Jiang and Dong [26]. Let *G* be the genotype matrix of a gene under investigation; *Q*_1_,*Q*_2_,*D* – vectors of two quantitative traits and a dichotomous phenotype respectively; *E*_*i*_, = 1,2,3 – vectors of age, gender and smoking status respectively; *R* – matrix of ten principal components obtained from Eigenstrat [27]. First, phenotypes, genotype and covariates were adjusted for population stratification as follows: adjusted genotype $\overset{⌣}{G}=G-RR^{T}G$; adjusted phenotypes ${\tilde{Q}}_{1}=Q_{1}-RR^{T}Q_{1},{\tilde{Q}}_{2}=Q_{2}-RR^{T}Q_{2},\tilde{D}=D-RR^{T}D$; adjusted covariates ${\tilde{E}}_{i}=E_{i}-RR^{T}E_{i},i=1,2,3$. Second, covariates were regressed out from adjusted phenotypes using the following models:

(1) $$\begin{array}{l} {\tilde{Q}}_{1}=a_{0}+\sum_{i=1}^{3}a_{i}{\tilde{E}}_{i}+q_{1};{\tilde{Q}}_{2}=b_{0}+\sum_{i=1}^{3}b_{i}{\tilde{E}}_{i}+q_{2};\\ \;\tilde{D}=c_{0}+\sum_{i=1}^{3}c_{i}{\tilde{E}}_{i}+d \end{array}$$

where *q*_1_,*q*_2_ and *d* are regression residuals. These residuals were tested for an association with adjusted genotype $\tilde{G}$. Statistical significance for each method was assessed using 1000 permutations. The power was calculated as a proportion of phenotype replicates significant at the type-1 error rate of 0.05.

### GAW17 analysis results: comparison with the score test

We considered three partitionings for our method: MAF partitioning (two groups: variants with MAF above and below 1%), functional partitioning (two groups: non-synonymous variants; synonymous and unknown variants), and combined partitioning (four groups defined by MAF and functionality). To assess the empirical type-1 error rate of our method and those of the score test we ran the analysis with randomly permuted residuals *q*_1_,*q*_2_ and *d* from (1). Additional file 3 shows the empirical type-1 error rates for *Q*_1_ and *Q*_2_ traits (Panel 1) and dichotomous phenotype (Panel 2). It should be noted that the estimate of type-1 error rate is distributed as an observed probability of success for a binomial random variable with the sample size of 200 (number of phenotype replicates) and the probability of success 0.05 (theoretical type-1 error rate). The double-sided 99% confidence interval for the estimate of type-1 error rate is 0.015–0.095. As can be seen from Additional file 3, the type-1 error was well controlled.

**Figure 3:**
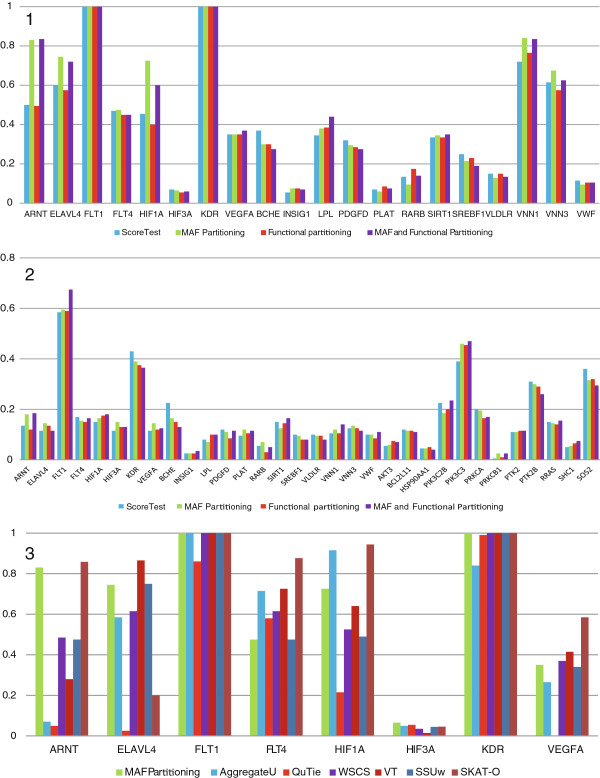
**Some results of GAW17 analysis.** Panel 1: Comparison of the proposed method (with different partitionings) with the score test for *Q*_1_ and *Q*_2_ causal genes and respective quantitative traits (*Q*_1_ causal genes are those from *ARNT* to *VEGFA*, *Q*_2_ causal genes are those from *BCHE* to *VWF*); Panel 2: Performance of the proposed method (with different partitionings) and the score test for the causal genes and a dichotomous trait; Panel 3: Comparison of the proposed method (MAF partitioning) with other methods on *Q*_1_ causal genes.

Panel 1 of Figure 3 shows the results for our method and the score test on genes that impacted *Q*_1_ and *Q*_2_ (genes from *ARNT* to *VEGFA* were tested on association with *Q*_1_, genes from *BCHE* to *VWF* – with *Q*_2_). Panel 2 of Figure 3 depicts the results of the analysis for the dichotomous trait. As can be seen, the score test was significantly more powerful for *KDR*, *BCHE* and *SOS2* genes (dichotomous phenotype), whereas for at least one partitioning the proposed method was substantially more powerful for *ARNT*, *ELAVL4*, *HIF1A* genes (*Q*_1_ trait); *VNN1* gene (*Q*_2_ trait); *FLT1* and *PIK3C3* genes (dichotomous phenotype). Table 1 shows our suggested reasons for the difference in performance between the score test and our proposed method for the genes listed above. As can be seen, for all the genes for which our method outperformed the score test, some of the considered partitionings contained a group consisting of only associated variants. This is likely to have resulted in our method achieving higher power compared with those of the score test. It should be noted that for *ELAVL4* gene the major association signal is borne by common variants, although according to the GAW17 phenotype model this gene contains only rare causal variants. To confirm this hypothesis, we performed an association analysis of *ELAVL4* common SNPs with *Q*_1_ trait using the score test. The power to identify an association was 79% after adjusting for population stratification and confounders. For the genes *KDR*, *BCHE* and *SOS2* we did not observe any clear separation of associated variants using any of the considered partitionings.

**Table 1:** Suggested reasons for the difference in power between the score test and our approach for some genes

**Genes**	**Partitioning**	**Suggested reason for the difference in performance**
***Genes for which our method outperformed the score test***		
*ARNT*	MAF and functionality	All common non-synonymous SNPs are causal.
*ELAVL4*	MAF	Association of the three common non-causal SNPs with *Q*_1_*.
*HIF1A*	MAF	The only common SNP is causal.
*VNN1*	MAF	The only common SNP is causal.
*FLT1*	MAF and functionality	All common non-synonymous SNPs are causal.
*PIK3C3*	MAF and functionality	All common non-synonymous SNPs are causal.
***Genes for which the score test outperformed our method***		
*KDR, BCHE, SOS2*	-	No clear separation of associated variants from neutral ones using any of the considered partitionings.

The table contains only those genes for which there was a significant difference in performance between the score test and our approach for any of the suggested partitionings.

*for details, see the subsection “GAW17 analysis results: comparison with the score test”.

### GAW17 analysis results: comparison with other tests

We contrasted the performance of our method (MAF partitioning) with those of WSCS [22], VT [18], SSUw [23] and SKAT-O with linear kernel and beta weights [24]. Also, we included some of the published results obtained by the participants of the GAW17 workshop: the performance of Multivariate Distance Matrix Regression (MDMR) and Mantel tests on *Q*_2_ causal genes [28], the performance of aggregated U-test (AggregateU) and CMC method (QuTie) on *Q*_1_ causal genes [29]. It should be noted that the adjustment for covariates and population stratification for SKAT-O test was done via the function “SKAT_Null_Model” of the R (http://cran.r-project.org) package “SKAT”. Also, in our implementation of the WSCS method rare variant weights were proportional to the absolute value of correlation between those rare variants and phenotype, since the original weights described by Dai et al. [22] were not applicable due to the adjusted phenotype being quantitative. Additional file 4 shows the empirical type-1 error estimates for WSCS, VT, SSUw and SKAT-O tests. Given that the 99% confidence interval for the empirical estimate of type-1 error rate is 0.015–0.095 as described previously, there was no evidence for type-1 error inflation.

Panel 3 of Figure 3 depicts the analysis results for *Q*_1_ causal genes. As can be seen, our method was among the top three most powerful approaches for *ANRT*, *ELAVL4* and *HIF1A* genes. Panel 1 of Additional file 5 shows the results for *Q*_2_ causal genes. Our method was the top performer for *VNN3* gene, and was among the top three performing approaches for *LPL* and *VNN1* genes. Panel 2 of Additional file 5 depicts the results of an association analysis of all causal genes with dichotomous phenotype. As can be seen, SKAT-O achieved the most notable power gains for *PIK3C3* and *PTK2B* genes. This emphasizes that SKAT-O may significantly outperform other tests under some phenotype models. However, this method also may significantly underperform other tests for some phenotype models, for example, *ELAVL4* gene with *Q*_1_ trait and *VNN1* gene with *Q*_2_ trait. Our proposed method was among the top three performing methods for *SOS2*, *PTK2B*, *PRKCA* genes and some other genes for which the power of all the methods was low.

## Discussion

In this article we have described a methodology that incorporates prior information into a region-based score test with the purpose of improving power to identify an association. Prior information, such as observed MAF, functional annotation, predicted deleteriousness of non-synonymous variants, may be used to partition variants within a region of interest. Then, this partitioning is utilized to construct a test statistic whose distribution under the null hypothesis has lower degrees of freedom compared with those of the score test. Based on the theoretical power comparison, population genetics simulations and GAW17 sequencing data analysis, we have shown that under some scenarios our method may perform as well as or outperform other methods.

Our suggested partitioning is splitting by functionality and if rare variants are present – by MAF with an arbitrary threshold, e.g., 1%. One of the major justifications for considering MAF in a partitioning design is that MAF may distinguish between different evolutionary forces acting on causal variants. Evolutionary theory predicts that variants that confer susceptibility to a disease which in turn reduces fitness are expected to have low MAF due to purifying selection. Empirically, using whole-exome sequencing data, it was found that non-synonymous substitutions are more significantly skewed towards low frequencies compared with synonymous variants. This finding “almost certainly reflects the operation of purifying selection” [30] acting on many genes across genome. Thus, if a causal gene is under strong purifying selection incorporating MAF into partitioning design is likely to be beneficial. On the other hand, a causal variant could have risen to higher frequency due to other forces such as balancing selection, mutation-selection balance, antagonistic pleiotropy, etc. [31]. Thus, for example, if a causal variant has been under a strong balancing selection it is likely to be common; and incorporating MAF into partitioning design may lead to power improvement. Partitioning by functionality may be beneficial for gene-based analysis in cases when an association signal comes from one or several functional groups of variants. For example, if highly deleterious non-synonymous variants within exons of a gene are causal, then partitioning by functionality is likely to improve power. Although the misclassification rate for prediction tools may be high (e.g., PolyPhen-2 achieved 92% power to detect truly damaging variants at 20% type-1 error rate over HumDiv data [32]), our method may still benefit from grouping variants by functional significance, since this is an effective way to give more emphasis on variants that are more likely to be causal [33]. The benefit of using functional information was demonstrated in several simulations and candidate gene sequencing studies [18,34,35].

There are a few limitations of the proposed methodology. First, it is unknown in advance which prior information is relevant for a given genomic region. Prior information that does not help to uncover groups of associated variants is likely to have negative impact on statistical power. Second, the test statistic (3) is, in general, not invariant with respect to the ordering of SNPs. This can be seen, for example, when the covariance matrix *V* is invariant with respect to permutation of elements of the score vector *U* (e.g., all the cross-SNP covariances are the same, and diagonal variances are equal). So, for any SNP ordering the Cholesky decomposition *A* is the same; hence, the test statistic is, in general, dependent on ordering. To clarify the ambiguity the ordering of SNPs according to a position on a chromosome may be assumed.

## Conclusions

A novel statistical approach that incorporates prior information in a region-based score test with the purpose of improving power has been proposed. Theoretical power comparison, population genetics simulations and the results of the GAW17 data set analysis showed that under some scenarios our method may perform as well as or outperform other methods.

## Methods

Consider a rare variants association study of a genomic region with a dichotomous or quantitative trait. Let us introduce the following notations: genotype matrix *G* = {*g*_*nl*_, *n* = 1, …, *N*, *l* = 1, …, *L*} coded as minor allele counts, where *n* and *l* are indices for individuals and variants respectively; *N* × 1 vector of genotypes for the *l*th variant *g*_*l*_ = {*g*_*nl*_, *n* = 1, …, *N*}; mean of genotype for the *l*th variant ${\bar{g}}_{l}$; phenotype vector *Y* = {*y*_*n*_, *n* = 1, …, *N*}, mean of phenotype $\bar{Y}$. Consider the generalized linear model *g*(*Ey*) = *b*_0_ + *Gb*, where *b* = {*b*_1_, …, *b*_*L*_} is a vector of regression coefficients, and *g* is a monotone link function. The score test statistic used to test an association of phenotype with genotype is as follows [36]:

(2) $$T_{0}=U^{T}V^{-1}U,$$

where $U={\left\{\sum_{n=1}^{N}\left(y_{n}-\bar{Y}\right)\left(g_{\mathrm{nl}}-{\bar{g}}_{l}\right)\right\}}_{l=1}^{L}$ is the vector of scores, $V=\mathrm{Cov}\left(G\right)\sum_{n=1}^{N}{\left(y_{n}-\bar{Y}\right)}^{2}$ is *L* × *L* the score covariance matrix assumed to be non-singular, and $\mathrm{Cov}\left(G\right)={\left\{\mathrm{Cov}\left(g_{l},g_{l'}\right)\right\}}_{l,l'=1}^{L}$ is the *L* × *L* genotype covariance matrix. Under the null hypothesis (none of the variants is associated with phenotype) *T*_0_ asymptotically follows chi-squared distribution with *rank*(*V*) degrees of freedom (d.f.). Under the alternative hypothesis (at least one variant is associated with phenotype) the asymptotic distribution of (2) can be approximated by a non-central chi-squared distribution.

Following is the description of our proposed method. First, let us transform the coordinates of score vector *U* to be asymptotically independent under the null hypothesis. If we denote *C* = (*A*^*T*^)^−1^, where matrix *A* is a Cholesky decomposition of *V* (*V* = *A*^*T*^*A*), the vector *S* = *CU*, under the null hypothesis, is asymptotically distributed as a standard multivariate normal random vector (since  *Cov*(*CU*) = *CCov*(*U*)*C*^*T*^ = (*A*^*T*^)^− 1^*VA*^− 1^ = (*A*^*T*^)^− 1^(*A*^*T*^*A*)*A*^− 1^ = *I*). It should be noted that under the alternative hypothesis S is approximately distributed as a multivariate normal random vector with nonzero mean and unit covariance matrix under the assumption of *V* being a reasonable approximation for the covariance matrix of *U*[37]. In general, any other decomposition of the covariance matrix *V* of the form *V* = *A*^*T*^*A* can be used. We apply Cholesky decomposition because it is fast to compute even for big covariance matrices.

Next, let us describe group statistics. Consider partitioning of *L* SNPs within a region into *K* disjoint groups *G*_*k*_, *k* = 1, …, *K* of size *L*_*k*_, *k* = 1, …, *K*. This partitioning is done using prior information, for example: by observed MAF (below and above some arbitrary frequency threshold); by functional annotation (non-synonymous and synonymous for exon sequencing studies; exonic, intronic, 3′UTR, 5′UTR and intergenic for gene-based studies); by functional significance for non-synonymous SNPS (benign, probably and possibly damaging from SIFT or PolyPhen output) etc. For each group let us define $t_{k}=\sum_{l\in G_{k}}s_{l}^{2},k=1,\ldots ,K$, where *s*_*l*_, *l* = 1, …, *L* is the *l*th coordinate of the vector *S*. It should be noted that partitioning based on prior to *S*, since *S* = (*A*^*T*^)^−1^*U* is the vector *U* in a new basis, which is the set of column vectors of the matrix *A*^*T*^ (old basis is a standard basis). Since the vector *S* is multivariate normal with unit covariance matrix, *t*_*k*_, *k* = 1, …, *K* are asymptotically independent. Under the null hypothesis *t*_*k*_ asymptotically follows central chi-squared distribution with *L*_*k*_ degrees of freedom, whereas under the alternative hypothesis at least one of the *t*_*k*_, *k* = 1, …, *K* is distributed as a non-central chi-squared random variable. Let us denote as *P*_*l*_ and *P*_*l*_^−1^ cumulative distribution function (CDF) of central chi-squared random variable with *l* d.f. and its inverse respectively. Group statistics *H*_*k*_ are defined as follows:

(3) $$H_{k}=P_{1}^{-1}\left(P_{L_{k}}\left(t_{k}\right)\right).$$

It should be noted that under the null hypothesis *H*_*k*_ follows a central chi-squared distribution with 1 d.f. Finally, the test statistic of our proposed method is:

(4) $$T_{1}=\sum_{k=1}^{K}H_{k}.$$

Under the null hypothesis *T*_1_ is distributed as a chi-squared random variable with *K* d.f. Large value of *T*_1_ indicates the deviation from the null hypothesis. Transformation (3) is used to decrease the degrees of freedom of the final test, as some of the groups *G*_*k*_, *k* = 1, …, *K* may contain only neutral variants. If *k*th group does not contain associated SNPs, group *G*_*k*_ contributes only 1 d.f. to the final test, whereas it adds *L*_*k*_ d.f. to the score test. Thus, under “informative” partitioning our proposed method may gain power compared with the score test.

There are few notes that should be taken into account when applying our method. First, the theoretical approximation for distribution of *t*_*k*_ may not hold under moderate sample sizes. Hence, in all our simulations and real data application we used the empirical CDF of *t*_*k*_, denoted as *F*_*k*_, obtained via 1000 permutations under the null hypothesis. Instead of $P_{L_{k}}\left(t_{k}\right)$ in (3) we calculated a Gaussian kernel CDF estimate *F*_*k*_(*t*_*k*_) with a multi-stage plug-in bandwidth of Polansky and Baker [38]. R (http://cran.r-project.org) package ‘kerdiest’ contains all the necessary algorithms. Second, the covariance matrix *V* may happen to be computationally or exactly singular when, for example, considering a region with high LD between common SNPs. In general, to avoid singularity issues we apply Monroe-Penrose pseudoinverse in (2) instead of *V*^−1^. This pseudoinverse is uniquely defined and equals the standard inverse matrix when *V* is non-singular. From the same considerations instead of usual Cholesky factoring we used generalized Cholesky decomposition, which is well defined for square symmetric positive semi-definite matrices. If *V* is non-singular, the generalized Cholesky decomposition equals the usual Cholesky factoring. Also, it should be noted that in general our test statistic is not invariant with respect to the ordering of variants within a region. To clarify the ambiguity we assume the ordering of SNPs according to a position on a chromosome (see the “Discussion” section).

### Theoretical power calculations

To evaluate our method we compared its theoretical power with those of the score test. Denote the non-centrality parameter (NCP) of the test statistic (2) under the alternative hypothesis as *r* (the connection between *r* and effect size is provided in the Additional files 6 and 7). Also, we will use the notations *P*_*l*,*r*_ and *P*_*l*,*r*_^−1^ for the CDF of non-central chi-squared random variable with *l* d.f. and NCP *r* and its inverse respectively. The power of the score test with type-1 error rate *α* is:

(5) $$1-{P_{L}}_{,r}\left(q_{1-\alpha ,L}\right),$$

where *q*_1−*α*,*L*_ is 1 − *α* quantile of the chi-squared distribution with *L* d.f. Let us assume that all the causal SNPs are uncorrelated with other SNPs within a region, and that they are in one of the *K* groups (without loss of generality, let it be the first group *k* = 1). Then, the power of the proposed test is:

(6) $$1-P\left(H_{1}+\chi_{K-1}^{2}<q_{1-\alpha ,K}\right),$$

since $\sum_{k=2}^{K}h_{k}$ follows a central chi-squared distribution with *K* − 1 d.f., because all associated SNPs are in the first group. The probability in (6) can be computed using the convolution of two CDFs as follows:

(7) $$P\;\left(H_{1}+\chi_{K-1}^{2}<q_{1-\alpha ,K}\right)=\int_{0}^{q_{1-\alpha ,K}}P\;\left(H_{1}<q_{1-\alpha ,K}-x\right)\;dP_{K-1}\left(x\right).$$

Under the alternative hypothesis non-centrality parameter of *T*_0_ and those of *t*_1_ are equal, since both statistics include all the associated SNPs within a region. Thus, the distribution of *H*_1_ is $P_{1}^{-1}\left(P_{L_{1}}\left(\chi_{L_{1},r}^{2}\right)\right)$, and the integrand in the equation (7) can be calculated as $P\left(H_{1}<q_{1-\alpha ,K}-x\right)=P_{L_{1},r}\left(P_{L_{1}}^{-1}\left(P_{1}\left(q_{1-\alpha ,K}-x\right)\right)\right)$. This allows us to compute the theoretical power of our method.

### Population genetics simulations

Boyko et al. [39] found a simple two-epoch expansion model to be one of the best-fit models for population genetics simulations of African-American genotype. Also, the authors found strong evidence of selective effects acting on new amino acid replacing mutations and inferred the distribution of those selective effects. The scaled population selection coefficient (defined as a selection coefficient multiplied by twice the effective population size) was assumed to follow a negative gamma distribution with the following parameters: shape 0.184 and scale 8200. Mutation rate was set at 1.8 × 10^−8^ per nucleotide per generation. The selective disadvantage was additive. Using the forward simulator SFS_CODE (http://sfscode.sourceforge.net) with the parameters described above, we generated 100 haplotype pools for a 3 kb coding genomic region. Each haplotype pool contained 4000 haplotypes. In our simulations we considered only non-synonymous variants. To generate a data replicate we first selected a haplotype pool at random. Within that pool we sampled a pair of haplotypes at random and added those haplotypes to form a multi-site genotype of an “individual”. Dichotomous phenotype was assigned based on this multi-site genotype using a linear logistic model through which we controlled the odds ratios of causal variants. The probability of a disease conditional on the wild type genotype was set at 1% for all phenotype models.

We considered four phenotype scenarios. For the first model called “Rare” randomly chosen 50% of rare variants (defined as those with MAF<1%) within a haplotype pool were assigned to be causal with uniform odds ratio of 3. For the “Low Frequency” scenario one randomly chosen low frequency SNP (MAF between 1% and 5% in a haplotype pool) was causal with odds ratio of 2.5. If there was no SNP with MAF between 1% and 5% in a haplotype pool, we selected a SNP with the lowest MAF above 5%. For the “Common” scenario one common SNP with MAF>5% in a haplotype pool was causal and had the odds ratio of 1.5. Finally, the “Interaction” scenario models the hypothesized interaction of common and rare variants in *RET* gene associated with Hirschsprung’s disease [40,41]. The simulation framework for this scenario was previously described by Liu and Leal [42]. Briefly, 50% of rare variants were assigned to be causal. Each causal rare minor allele increased the odds of a disease by 6 times if and only if it was present on the same haplotype as a minor allele of a common SNP randomly chosen beforehand.

Sample sizes were the following: 500 cases and 500 controls for “Rare”, “Low frequency” and “Common” scenarios; 1000 cases and 1000 controls for “Interaction” model. In total 1000 data replicates were generated for each scenario. Power was estimated as a proportion of data replicates significant at a fixed type-1 error of 0.05. For all the statistical tests 1000 permutations were applied to estimate a significance level.

## Competing interests

The authors declare that they have no competing interests.

## Authors’ contributions

SZ, AS and AT conceived the study. SZ and AT designed the experiments. SZ and GHKT conducted the experiments and performed the analysis. SZ wrote the manuscript. All authors read and approved the final manuscript.

## Supplementary Material

Additional file 1 **The difference in theoretical power (vertical axis) between the proposed test and the score test as a function of the total non-centrality parameter *r* (horizontal axis) at the genome-wide type-1 error rate *α* = 0.05/35000. Each curve corresponds to the number of SNPs in the causal group *L***_***1***_** given in the legend (Panels 1 and 2), number of groups *K* (Panel 3), and number of causal groups *m* (Panel 4).** The parameters for each of the Panels are as follows: Panel 1: *L* = 10, *K* = 2; Panel 2: *L* = 100, *K* = 10; Panel 3: *L* = 50, *L*_1_ = 5; Panel 4: *L* = 54, *K* = 6, equal number of SNPs in each group, and equal non-centrality parameters in all causal groups. Click here for file

Additional file 2 **The estimate of empirical type-1 error rate of the proposed method with MAF partitioning, the score test, VT, WSCS, SSUw and SKAT-O for population genetics simulations.** The theoretical type-1 error was assumed to be 0.05. The data for the estimate of the type-1 error was generated using the null phenotype model: no association of genotype with phenotype. Click here for file

Additional file 3 **The estimate of empirical type-1 error rate of the proposed method with different partitionings and those of the score test for the causal genes in GAW17 data.** The theoretical type-1 error was assumed to be 0.05. Panel 1: *Q*_1_ and *Q*_2_ causal genes and respective quantitative trait (*Q*1 causal genes are those from *ARNT* to *VEGFA*, *Q*_2_ causal genes are those from *BCHE* to *VWF*); Panel 2: causal genes and dichotomous trait. Click here for file

Additional file 4 **The estimate of empirical type-1 error rate of WSCS, VT, SSUw and SKAT-O tests for the causal genes in GAW17 data.** The theoretical type-1 error was assumed to be 0.05. Panel 1: *Q*_1_ and *Q*_2_ causal genes and respective quantitative trait (*Q*_1_ causal genes are those from *ARNT* to *VEGFA*, *Q*_2_ causal genes are those from *BCHE* to *VWF*); Panel 2: causal genes and a dichotomous trait. Click here for file

Additional file 5 **Some results of GAW17 analysis.** Panel 1: Comparison of the proposed method (MAF partitioning) with other methods on *Q*_2_ causal genes; Panel 2: Comparison of the proposed method (MAF partitioning) with other methods on causal genes and a dichotomous phenotype. Click here for file

Additional file 6 **The link between the non-centrality parameter and the effect size for the region-based score test.** The derivation of the equation connecting the non-centrality parameter (NCP) and the effect size for a region-based score test, and the description of assumptions for illustrating the dependence of NCP on the effect size in Additional file 7. Click here for file

Additional file 7 **The non-centrality parameter (vertical axis) as a function of the effect size (relative risk) of each causal variant (horizontal axis) under the assumptions described in Additional file **6**.** Curves within each panel correspond to the number of causal variants within the region. The assumptions are as follows: all variants within a region are independent; all the causal variants have the same MAF and the same effect size; 500 cases and 500 controls. The MAF of the causal variants are as follows: Panel 1 – 1%; Panel 2 – 0.5%; Panel 3 – 0.25%: Panel 4 – 0.125%. Click here for file
