# Supplementary figures and images for: A method to incorporate prior information into score test for genetic association studies
Source: BMC Bioinformatics. 2014 Jan 22;15:24. doi: 10.1186/1471-2105-15-24 (PMC3904928; doi:10.1186/1471-2105-15-24)

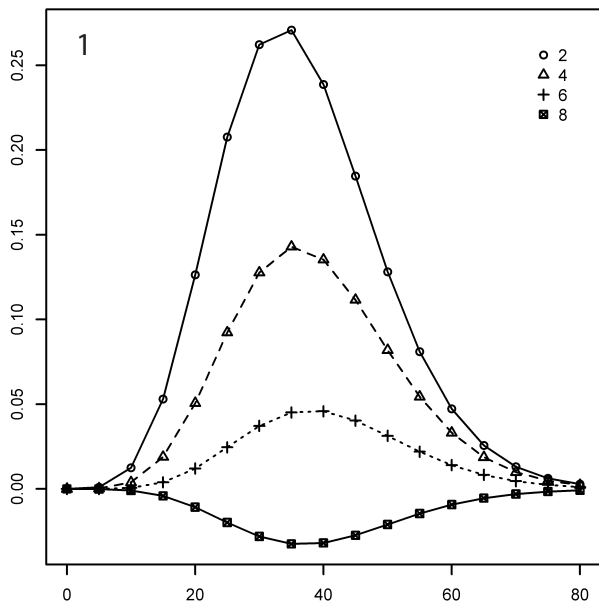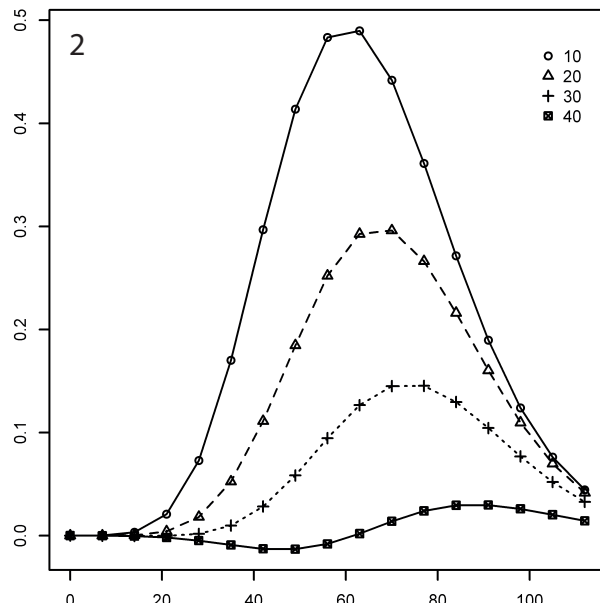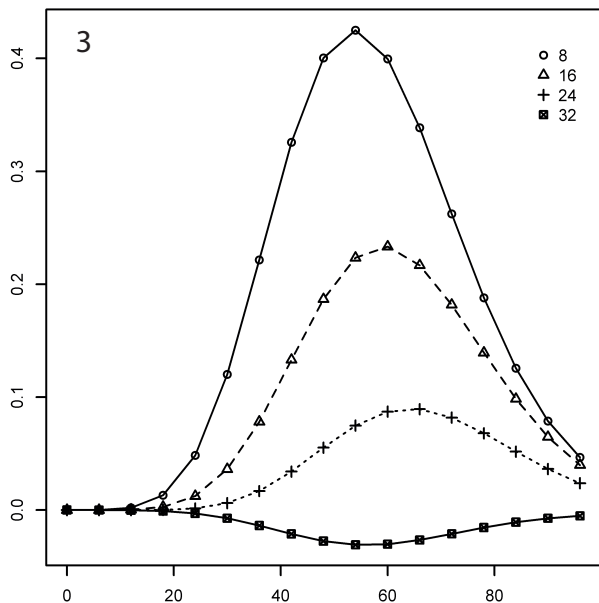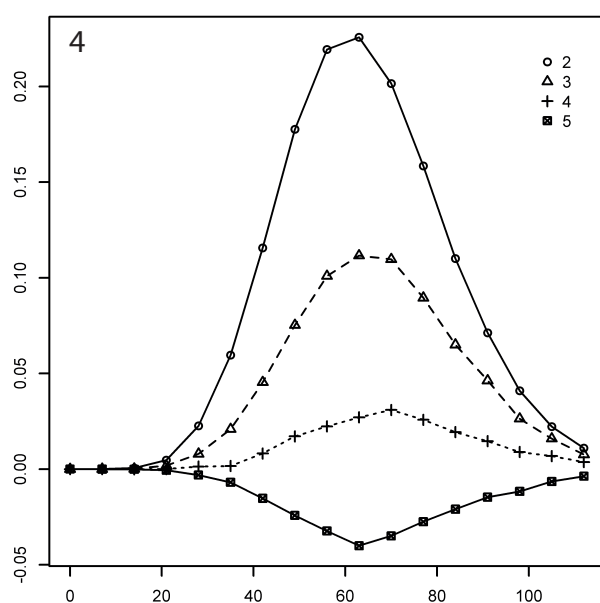

Supplement: Additional file 1 — The difference in theoretical power (vertical axis) between the proposed test and the score test as a function of the total non-centrality parameter r (horizontal axis) at the genome-wide type-1 error rate α = 0.05/35000. Each curve corresponds to the number of SNPs in the causal group L 1 given in the legend (Panels 1 and 2), number of groups K (Panel 3), and number of causal groups m (Panel 4). The parameters for each of the Panels are as follows: Panel 1: L = 10, K = 2; Panel 2: L = 100, K = 10; Panel 3: L = 50, L1 = 5; Panel 4: L = 54, K = 6, equal number of SNPs in each group, and equal non-centrality parameters in all causal groups. [file 1471-2105-15-24-S1.pdf]

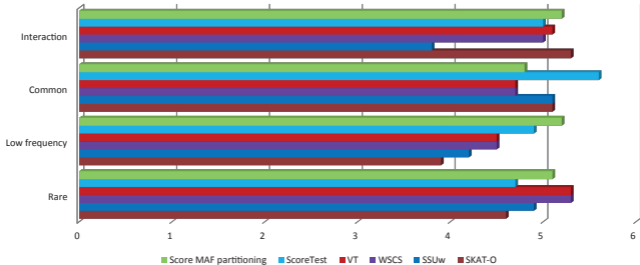

Supplement: Additional file 2 — The estimate of empirical type-1 error rate of the proposed method with MAF partitioning, the score test, VT, WSCS, SSUw and SKAT-O for population genetics simulations. The theoretical type-1 error was assumed to be 0.05. The data for the estimate of the type-1 error was generated using the null phenotype model: no association of genotype with phenotype. [file 1471-2105-15-24-S2.pdf]

1

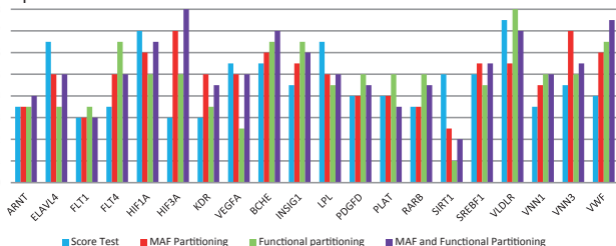

2

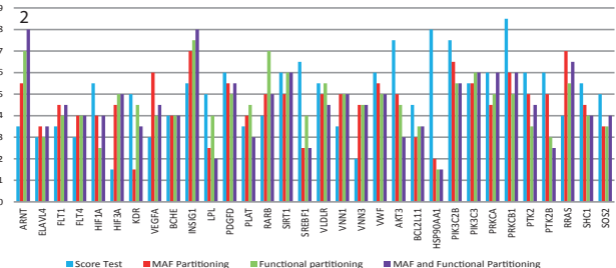

Supplement: Additional file 3 — The estimate of empirical type-1 error rate of the proposed method with different partitionings and those of the score test for the causal genes in GAW17 data. The theoretical type-1 error was assumed to be 0.05. Panel 1: Q1 and Q2 causal genes and respective quantitative trait (Q1 causal genes are those from ARNT to VEGFA, Q2 causal genes are those from BCHE to VWF); Panel 2: causal genes and dichotomous trait. [file 1471-2105-15-24-S3.pdf]

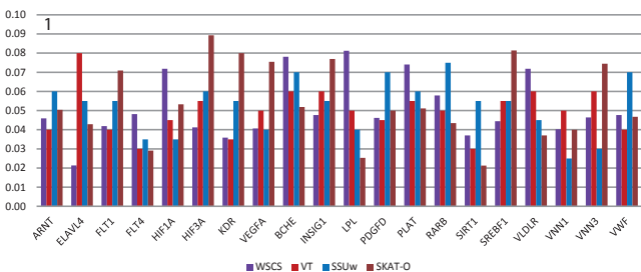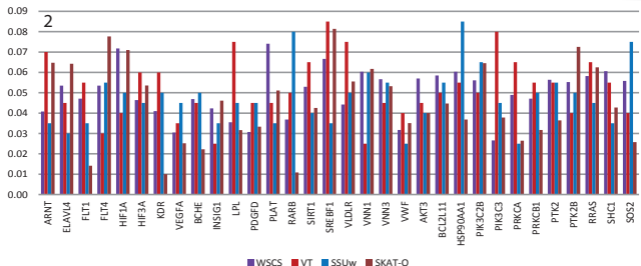

Supplement: Additional file 4 — The estimate of empirical type-1 error rate of WSCS, VT, SSUw and SKAT-O tests for the causal genes in GAW17 data. The theoretical type-1 error was assumed to be 0.05. Panel 1: Q1 and Q2 causal genes and respective quantitative trait (Q1 causal genes are those from ARNT to VEGFA, Q2 causal genes are those from BCHE to VWF); Panel 2: causal genes and a dichotomous trait. [file 1471-2105-15-24-S4.pdf]

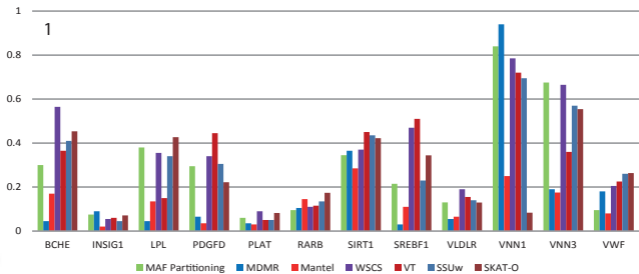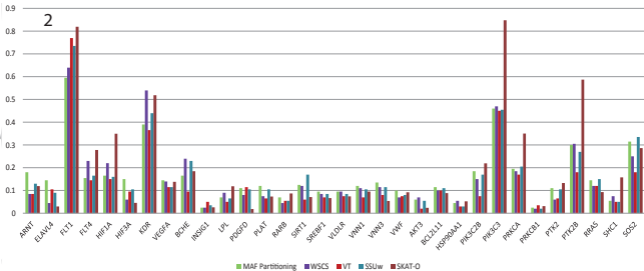

Supplement: Additional file 5 — Some results of GAW17 analysis. Panel 1: Comparison of the proposed method (MAF partitioning) with other methods on Q2 causal genes; Panel 2: Comparison of the proposed method (MAF partitioning) with other methods on causal genes and a dichotomous phenotype. [file 1471-2105-15-24-S5.pdf]

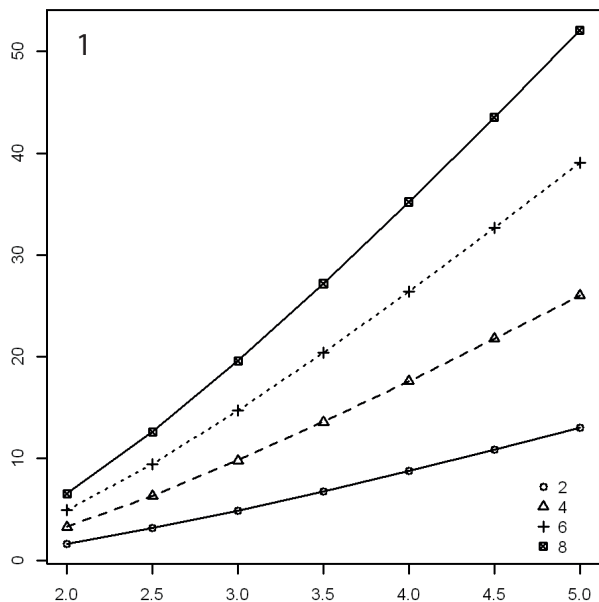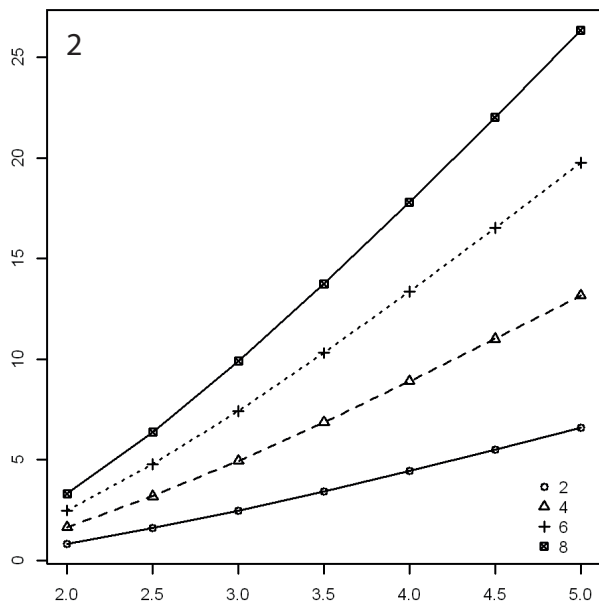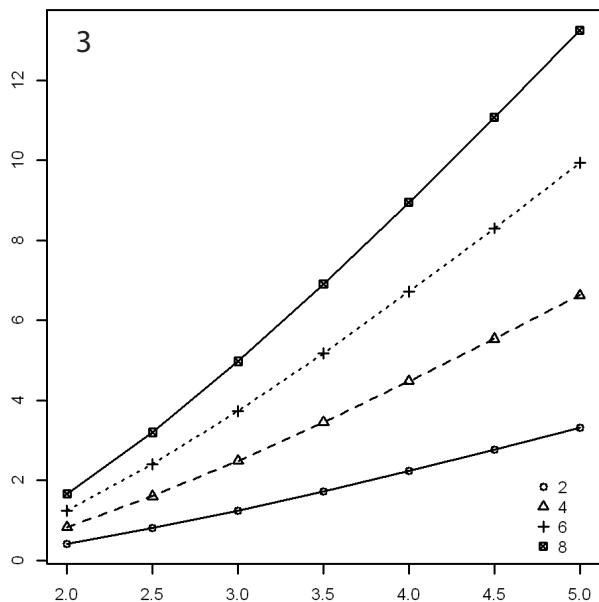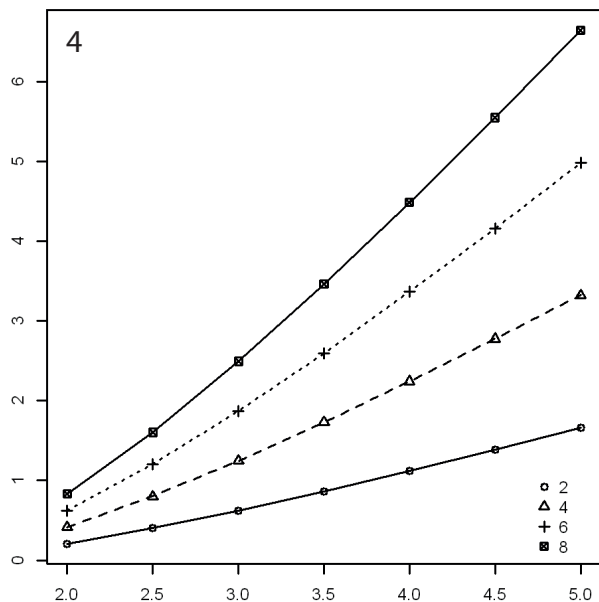

Supplement: Additional file 7 — The non-centrality parameter (vertical axis) as a function of the effect size (relative risk) of each causal variant (horizontal axis) under the assumptions described in Additional file 6. Curves within each panel correspond to the number of causal variants within the region. The assumptions are as follows: all variants within a region are independent; all the causal variants have the same MAF and the same effect size; 500 cases and 500 controls. The MAF of the causal variants are as follows: Panel 1 – 1%; Panel 2 – 0.5%; Panel 3 – 0.25%: Panel 4 – 0.125%. [file 1471-2105-15-24-S7.pdf]
