# Supplementary material for: A method to incorporate prior information into score test for genetic association studies
Source: BMC Bioinformatics. 2014 Jan 22;15:24. doi: 10.1186/1471-2105-15-24 (PMC3904928; doi:10.1186/1471-2105-15-24)
Supplement: Additional file 6 — The link between the non-centrality parameter and the effect size for the region-based score test. The derivation of the equation connecting the non-centrality parameter (NCP) and the effect size for a region-based score test, and the description of assumptions for illustrating the dependence of NCP on the effect size in Additional file 7. [file 1471-2105-15-24-S6.docx]

**Link between non-centrality parameter and effect size for the region-based score test**

Adopting the notations as in the article, let us consider the element of the score vector $U_{l}=\sum_{n=1}^{N} \left( y_{n}-\bar{Y} \right)\left( g_{nl}-\bar{g_{l}} \right), l=1,\ldots,L$. Denote the number of cases, controls and total sample size as $N^{A}, N^{U}$ and $N=N^{A}+N^{U}$. After algebraic transformations it can be shown that:

|  | $U_{l}=2N^{A}N^{U}\left( f_{l}^{+}-f_{l}^{-} \right)/N$ | (1) |
| --- | --- | --- |

where $f_{l}^{+}$ and $f_{l}^{-}$ are the observed MAF in cases and controls, respectively, for the $l$th SNP. Then, the vector $S=CU$ is asymptotically distributed as a multivariate random vector with the unit covariance matrix and mean $CE(U)$, where $E(U)$ is the mathematical expectation of the score vector, which can be written as follows:

|  | $E\left( U \right)=E\left( \left\{ U_{l} \right\}_{l=1}^{L} \right)=\left\{ 2N^{A}N^{U}({ef}_{l}^{+}-{ef}_{l}^{-})/N \right\}_{l=1}^{L},$ | (2) |
| --- | --- | --- |

where $ef_{l}^{+}$ and $ef_{l}^{-}$ are population MAF in cases and controls of the $l$th variant. If we denote as $R_{l}$ relative risk of the $l$th SNP and assume low prevalence of a disease it follows [[1](#_ENREF_1)]:

|  | ${ef}_{l}^{+}=\frac{R_{l}{ef}_{l}^{-}}{\left( 1+\left( R_{l}-1 \right){ef}_{l}^{-} \right)}.$ | (3) |
| --- | --- | --- |

The score test statistic is the sum of squares of elements of vector $S$. If we define vector $\xi=\left\{ \xi_{l} \right\}_{l=1}^{L}=E\left( S \right)=CE(U)$, then the non-centrality parameter (NCP) of the score test statistic under the alternative hypothesis is:

|  | $r=\sum_{l=1}^{L} \xi_{l}^{2}$ | (4) |
| --- | --- | --- |

Under the null hypothesis of no variant being associated with a phenotype, which is equivalent to $R_{l}=1, l=1,\ldots,L$, it follows from (3) that $ef_{l}^{+}=ef_{l}^{-}$, which implies from (2) $E\left( U_{l} \right)=0, l=1,\ldots,L$ and $\xi=CE\left( U \right)=\left\{ 0 \right\}_{l=1}^{L}$; thus, $r=0$.

**Description of the assumptions for illustration of connection between non-centrality parameter and effect size**

From the considerations above, it can be seen that NCP $r$ is a function of the number of cases $N^{A}$ and controls $N^{U}$in the study, relative risk of each variant $R_{l}, l=1,\ldots,L$; population MAF in controls $ef_{l}^{-}, l=1,\ldots,L$; and covariance matrix of the score test statistic $V$ (since matrix $C=\left( A^{T} \right)^{-1},$ where $V=A^{T}A$). To illustrate the dependence between NCP and relative risk let us assume independence of variants within the region, which implies the matrix $V$ is diagonal. Thus, $V=diag\left( \left\{ v_{l} \right\}_{l=1}^{L} \right)$ where $v_{l}=var\left( g_{l} \right)$ is variance of the $l$th SNP in our sample. It follows that $var\left( g_{l} \right)=2N^{U}\left( 1-ef_{l}^{-} \right)ef_{l}^{-}+2N^{A}\left( 1-ef_{l}^{+} \right)ef_{l}^{+},$ which is variance of the sum of two independent binomial random variables with the number of draws $2N^{U}$ and $2N^{A}$ and the probability of success $ef_{l}^{-}$ and $ef_{l}^{+}$ respectively. It follows:

|  | C = $diag(1/\sqrt{2N^{A}\left( 1-ef_{l}^{+} \right)ef_{l}^{+}+2N^{U}\left( 1-ef_{l}^{-} \right)ef_{l}^{-}} ,l=1,\ldots,L)$. | (5) |
| --- | --- | --- |

So, given population MAF of causal variants in controls $ef_{l}^{-}$, relative risk of causal variants $R_{l}$, the number of cases $N^{A}$ and controls $N^{U}$, we can calculate the corresponding NCP $r$ according to the following algorithm:

1. calculate $ef_{l}^{+}$ – population MAF in cases from (3)
2. calculate $E(U)$ – the expectation of the score vector $U$ from (2)
3. obtain matrix $C$ from (5)
4. calculate vector $\xi=\left\{ \xi_{l} \right\}_{l=1}^{L}=CE(U)$
5. obtain NCP $r$ from (4).

For the purpose of illustration, let us assume $N^{A}=N^{U}=500$, population MAF and relative risk of all causal variants are equal. Additional File 7 depicts the non-centrality parameter (vertical axis) as a function of relative risk (horizontal axis) and the number of causal variants (lines within each panel). Population MAF of causal variants in controls was the following: Panel 1 – 1%, Panel 2 – 0.5%, Panel 3 – 0.25%, Panel 4 – 0.125%. As can be seen, the non-centrality parameter monotonically increases with increasing relative risk, population MAF in controls and the number of causal variants within a region.

**References**

1. Sul JH, Han B, He D, Eskin E: **An optimal weighted aggregated association test for identification of rare variants involved in common diseases**. *Genetics* 2011, **188**(1):181-188.
